# Impact of Obesity on Hospitalization Burden of Urolithiasis in Spain Between 1997 and 2021

**DOI:** 10.3390/jcm14020381

**Published:** 2025-01-09

**Authors:** María Rodríguez-Monsalve, Manuel Durán-Poveda, Victoria Gómez Dos Santos, Javier Burgos Revilla, Elena García-Criado, Dolores Prieto, Ángel Gil de Miguel, Javier Sáenz-Medina

**Affiliations:** 1 Department of Urology, Puerta de Hierro-Majadahonda University Hospital, 28222 Madrid, Spain; 2 Department of Medical Specialties and Public Health, King Juan Carlos University, 28933 Madrid, Spain; 3 Department of Urology, Ramón y Cajal University Hospital, 28034 Madrid, Spain; 4 Department of Physiology, Pharmacy School, Complutense University, 28040 Madrid, Spain

**Keywords:** urolithiasis, obesity, metabolic syndrome, epidemiology

## Abstract

**Background/Objective**: The prevalence of kidney stones has increased, especially in industrialized countries. Obesity and metabolic syndrome have also risen significantly and are considered factors driving this trend. Our goal was to assess the hospitalization burden of kidney stones and identify epidemiological trends in Spain over the past 25 years. Additionally, this study analyzed the relationship between the data of kidney stone patients and obesity-coded diagnoses in hospital discharges. **Methods**: A retrospective observational study was conducted with the data from 1,070,192 patients hospitalized for urolithiasis between 1997 and 2021 using the Minimum Basic Data System. The hospitalization burden of lithiasis and its association with obesity was analyzed, alongside trend evaluations. The incidence of lithiasis in obese hospitalized patients versus all hospitalizations was compared. Mortality rates, length of stay, and the costs of lithiasis hospitalizations were evaluated to determine obesity’s impact on lithiasis patients. **Results**: Kidney stone cases increased by 1.8% annually (CI 95%: 0.2–3.3), with a more pronounced 10.7% (CI 95%: 7.3–14.2) increase in obesity-related hospitalizations. Obesity increased the likelihood of lithiasis hospitalization by 15.6 times and was associated with higher hospitalization costs. However, obesity did not significantly affect hospital stay duration or mortality rates. **Conclusions**: The increasing burden of kidney stone hospitalizations in Spain is linked to rising obesity rates. Obesity contributes to higher hospitalization costs. Preventive strategies targeting obesity should be implemented to reduce the burden on healthcare systems.

## 1. Introduction

The incidence and prevalence of kidney stones have been reported in several publications showing an increasing trend. The overall probability of forming stones differs worldwide, with it being 1–5% in Asia, 5–9% in Europe, 13% in North America, and 20% in Saudi Arabia [1]. Furthermore, differences in terms of incidence, distribution, anatomical stone localization, or stone composition have been reported across the world. Several factors have been pointed to explain this trend such as race ethnicity, diet, and climatic factors [2].

The prevalence of kidney stones has been reported to have increased worldwide since the studies in 1965–1970 to nowadays. Stone prevalence increased with increasing age in countries such as Germany, Iceland, Iran, Italy, Greece, Turkey, and the United States. Although the prevalence of kidney stones varies across different regions, it is observed that more men tend to develop stones compared to women, with sex ratios ranging from 2.5:1 in Japan to 1.15:1 in Iran. Also, it is generally believed that in developed countries about 1–2% of patients with stones are children [3]. The cause of the increasing prevalence and incidence of kidney stones is unclear, and genetic and environmental factors have been implied. However, dietary factors and climate have been pointed out to have a major impact on these trends [3].

On the other side, obesity has become a public health problem. A new study released in The Lancet shows that in 2022 more than 1 billion people in the world are now living with obesity. Worldwide, obesity among adults has more than doubled since 1990 and has quadrupled among children and adolescents (5–19 years of age). The data also show that 43% of adults were overweight in 2022 [4].

There is historical evidence of the influence of the diet on stone formation. Animal protein intakes, the ingestion of fast foods, high fructose, and increased sodium consumption have been demonstrated to promote stone formation and influence the stone composition [5]. Furthermore, kidney stones have been associated with other metabolic disturbances such as diabetes, being overweight, and metabolic syndrome [6].

In Spain, there is a lack of official data estimating the epidemiology of kidney stones. An incidence and prevalence of 0.7% and 5.06%, respectively, have been estimated in previous reports. Renal stones predominantly affected 40–60-year-old patients, being similar between both genders. Dietary aspects and especially animal protein intake have been pointed out as influent factors for calcium oxalate and uric acid stone formers [7,8].

The aim of this study was to evaluate the hospital burden of urolithiasis from 1997 to 2021 and to analyze the epidemiological trends during this period. Additionally, this study examines the relationship between the data from kidney stone patients and obesity-coded diagnoses in hospital discharges, both among patients with urolithiasis and in the general population. This analysis seeks to determine the impact of obesity on the incidence of hospitalizations for urolithiasis.

## 2. Materials and Methods

### 2.1. Study Design

A retrospective observational study was performed in Spain with data from the period between 1997 and 2021. The data were obtained from the Minimum Basic Data System (MBDS) that covers over 99% of the Spanish population. These records from public and private clinics provide information on 98% of hospitalization diagnostic codes from the discharge database of the Spanish Ministry of Health, according to the ICD-9-CM and 10 coding for diagnoses and procedures. Access to the MBDS was provided by Subdirección General del Instituto de Información Sanitaria. The population data were obtained from the National Institute of Statistics (INE).

From 1997 to 2015, the data were collected according to ICD-9- CM and from 2016 to 2021 using ICD-10-CM; all records of ureteral stones, kidney stones, and hydronephrosis with renal and ureteral stone obstruction as the diagnostic code were included as well as obesity. Additional variables such as age, gender, length of stay, mortality, and costs were included. Rates of hospitalization were calculated per 100,000 inhabitants according to the information of the National Institute of Statistics for the population (https://www.ine.es/jaxiT3/Tabla.htm?t=56934, accessed on 12 November 2024). All the information used by the researchers was coded and anonymous.

### 2.2. Statistical Analysis

An analysis of the hospitalization burden of lithiasis patients and obesity related to the general population was performed with a 95% confidence interval. The data were presented as persons per 100,000 inhabitants.

To establish the influence of obesity on lithiasis hospitalization burden incidence, the incidence of lithiasis in obesity-hospitalized patients vs. lithiasis diagnosis coded in all hospitalizations was compared, related to the general population through a chi square test. Additionally, the odds ratio stratified by age was calculated.

For the trend analysis, the five-year average percent change was calculated using the join–point regression model. Statistical differences were achieved when *p* < 0.05 and 95% confidence intervals were represented.

The impact of obesity on mortality, length of stay, and cost in normal weight and obese patients was also calculated using t student (length of stay and costs) and chi square tests (mortality) stratified by age, showing significance when the *p* value was <0.05.

## 3. Results

### 3.1. Demographic and Basal Characteristics

A total of 1,070,192 hospitalizations were performed with diagnostic codes of kidney or ureteral stones from 1997 to 2021 in Spain. The mean age of the patients was 58.8 years (CI 95%: 58.8–58.8); 603,103 were male (56.4% CI 95%: 56.3–56.4), 467,000 were women (43.6%, CI 95%: 43.5–43.7), and 89 hospitalizations did not have the gender recorded. Patients were hospitalized for an average time of 7.45 days (CI 95%: 7.41–7.49). The cost of hospitalization was only collected from 2016 onward. The average cost was EUR 4072 per hospitalization episode (CI 95%: 4059–4086). A total of 32,817 patients (3.1%, CI 95%: 3–3.1) died during hospitalization.

On the other hand, 3,416,068 hospitalizations were coded with a diagnosis of obesity, both as a primary or a secondary diagnosis, in Spain during the 25 years previously mentioned. The average age was 65.1 years (CI 95%: 65.1–65.1); however, in this group, females were predominant over males, 57.7% (CI 95%: 57.7–57.8) vs. 42.3% (CI 95%: 42.2–42.3). The average hospital stay of this group was 8.4 days (CI 95%: 8.4–8.4), and the average cost of admission was EUR 5595 (CI 95%: 5586–5604). A total of 145,416 patients (4.3%, CI 95%: 4.2–4.3) of the total obese hospitalized patients died.

### 3.2. Trends of Lithiasis and Obesity in Spanish Hospitalizations

An analysis of five-year rates of hospitalization burden of urolithiasis showed an increase from 1997 to 2021. The data are reported in Table 1. The mean overall hospitalization burden of stone disease for these periods was 95.6 hospitalizations per 100,000 habitants (CI 95%: 95.4–95.8).

The percentage change in hospital admissions stratified by age from 1997 to 2021 was estimated and is detailed in Table 1 showing an increase that reached statistical significance from the third to seventh decade of age, although the third decade showed the highest increase. These findings were significant in the total population with a mean percentage change of 1.8% (CI 95%: 0.2–3.3). According to these findings, trends in the hospitalization burden for all causes of lithiasis are on a slight rise.

The same examination of the five-year rate of hospitalization burden for obesity for the period of 1997–2021 was also conducted. The data of this analysis are shown in Table 2. The mean overall hospitalization rate coded for obesity was 305.2 hospitalizations per 100,000 habitants (CI 304.9–305.5). The five-year percentage change calculated in every age range was significant varying from 8.1% (CI 5.8–10.4) in the 50–59 years group to 16.1% (CI 11.4–20.9) in the 20–29 years one.

These data reflect an important increase in the obesity code in Spanish hospitalizations from 1997 to 2021.

**Figure 1:**
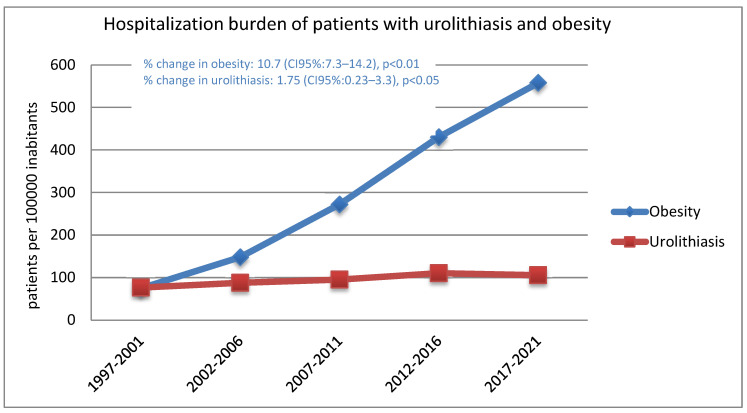
Rates of hospitalization burden of patients with renal or kidney stones and obesity between 1997 and 2021. The data are represented as the rate per 100,000 inhabitants. The percentage change is calculated by join–point regression estimation.

To reflect the percentage change in the hospitalization burden in both categories (lithiasis and obesity) Figure 1 was constructed. The quinquennial percentage change in the rates of hospitalization for lithiasis and obesity are both represented. Even though a significant percentage change (*p* < 0.05) is demonstrated in both pathologies, the changes are more noticeable in the hospitalization rates for obesity where a 10.7% change (CI 95% 7.3–14.2) was calculated.

**Figure 2:**
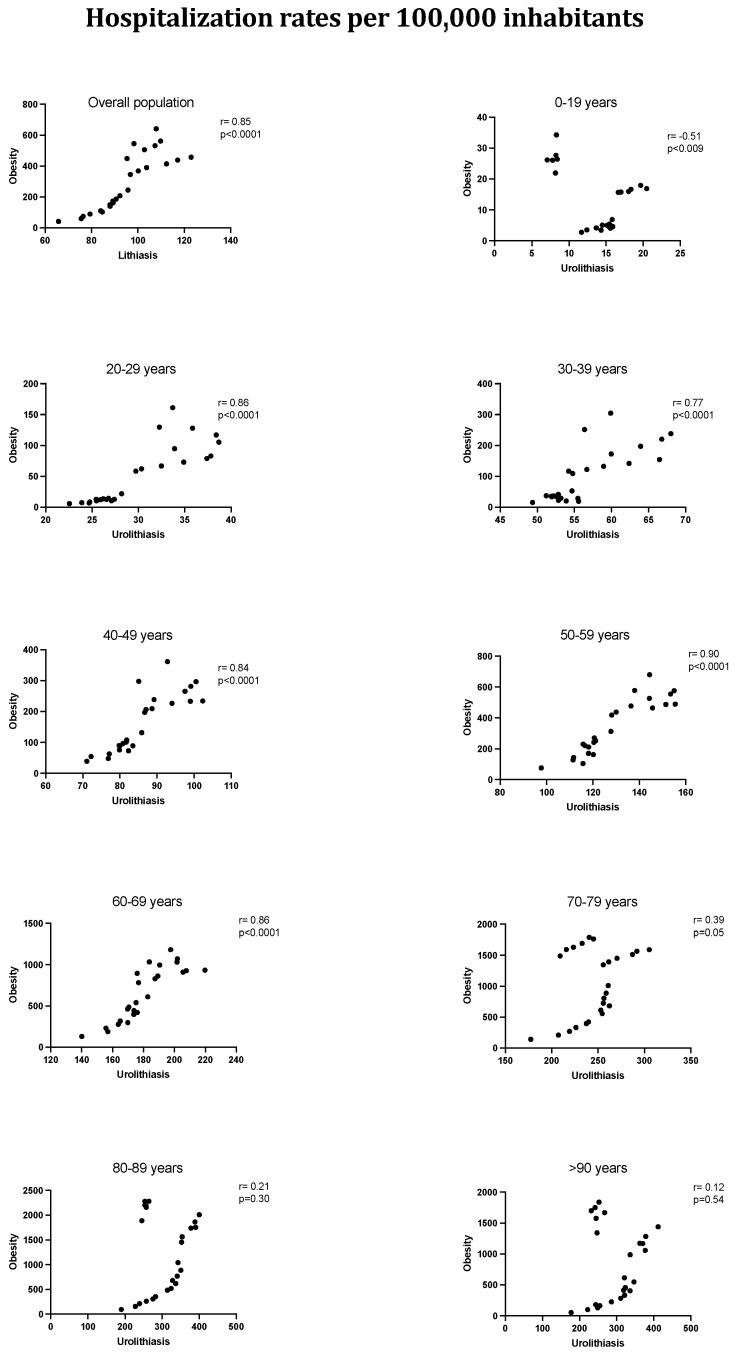
Annual rates of hospitalization burden of obesity and urolithiasis between 1997 and 2021 (*p* value for correlation between both rates).

A correlation test was also performed between the hospitalization burden of patients with kidney stones and the hospitalization burden of patients with an obesity diagnosis to establish a relationship between obesity and lithiasis incidence. It is remarkable that an important correlation was found between both populations (r = 0.85) with a significative statistical signification (*p* < 0.0001). When stratified by decades of age, the statistical signification has been maintained from the earliest decades of age to the seventh decade. The oldest patients did not reach signification, pointing out the relationship between kidney stones and obesity in young and middle-aged patients (Figure 2).

### 3.3. Hospitalized Lithiasis Incidence in the General and Obese Populations

The data collected in the MBDS cover almost all the hospitalization reports in Spain (>99%), so they can be used to consider the total burden of hospitalization per 100,000 habitants in the different codes.

To measure the influence of obesity in the development of lithiasis hospitalization events, we aimed to compare the hospitalized lithiasis incidence related to the general population with the one in the obese population. In Table 3, the results of this comparison are shown, and it proves that the obese condition multiplies by 15.6 the possibility of having a lithiasis event compared to the general population. This relationship is even strongest in the youngest population; the odds ratio in the <20 years population increases up to 46.4. The risk gradually decreases with age. In the elderly population over 89 years old, obesity only increases the risk of a lithiasis event with hospitalization by 2.3 times, but it remains significant.

### 3.4. Influence of Obesity on Mortality, Length of Stay, and Costs of Hospitalized Lithiasis Patients

It is well established that people affected by obesity have a greater risk of presenting comorbidity and developing subsequent hospitalization-related complications, compared with healthy individuals [9]. We aimed to study the differences in mortality, length of stay, and costs in both obese and normal-weight hospitalized lithiasis populations. The results are expressed in Table 4. Obesity does not have a significant impact on the length of stay of hospitalized stone formers. The mean length of stay in these situations was 7.4 days in normal-weight patients versus 7.5 days in obese patients. Excluding children, the length of stay had an increasing tendency in both populations, rising with age. This condition can be related to the increased comorbidities that appear in older people.

Mortality rates during hospitalization for lithiasis causes is 3% in the normal weight group and 2.6% per 100,000 inhabitants in the obesity group. Differences only appear in the population over 50 years old, higher for the normal-weight population. This might be because there are other relevant associated risk factors that have not been considered; nevertheless, the obesity condition does not seem a risk factor for mortality in lithiasis patients. Hospitalization costs were studied for the period of 2016 to 2021 (previous data were not available) and show a statistically significant difference between the obese and normal-weight population. The mean cost of hospital stay in the normal-weight population was EUR 4015, with this amount increasing up to EUR 4789 in the obesity group; this trend remains in all the central age populations. Only in populations of extreme ages (<20 years and >80 years) is the difference not statistically significant. This issue might be related to the smallest number of registers because, although differences were not reached, the mean costs of hospital stays remain higher for the obese group of hospitalizations.

## 4. Discussion

The prevalence of urolithiasis has increased in the last few decades affecting a wide spectrum of the population worldwide [10]. These rates ranged from 7 to 13% in North America, 5 to 9% in Europe, and 1 to 5% in Asia. Increased stone prevalence has been attributed to population growth and also to the increased prevalence of obesity and diabetes [1,11].

Obesity is an expanding condition, with data showing an incredible increase. The number of overweight people, defined as individuals with a body mass index (BMI) greater than 25 kg/m^2^, was projected to rise from 1.6 million in 2005 to 3.3 million in 2015. Additionally, the number of obese individuals, defined as those with a BMI greater than 30, was expected to increase from 400 million to 700 million over the same period [12].

Nowadays, lithiasis disease is considered a multifactorial condition. Etiological risk factors can be divided into two categories: the ones related to urinary composition and those with morphoanatomical factors. It is well established nowadays that the composition, macrostructure, and microstructure of renal stones are related to the etiological factors that are involved in their formation [13,14].

Obesity and environmental and dietary factors have been pointed out as possible causes that explain the increasing kidney stone prevalence. The influence of animal protein consumption on the whole stone-forming risk and the chemical composition of urinary calculi is well established [1]. Obesity, diabetes, hypertension, and metabolic syndrome are also considered risk factors for stone formation, and conversely, stone formers present a higher risk of hypertension and chronic kidney disease. The increasing prevalence of these metabolic disturbances, along with environmental factors such as global warming, have been reported as the causes of the increasing prevalence over the past 50 years and the expected future increases [15].

Epidemiological approaches have widely shown that metabolic syndrome and obesity increase the risk of developing kidney stones between 1.5 and 2.2 times [16,17,18]. Additionally, diabetes also has been associated with kidney stones increasing the risk by about 1.31 times in men and 1.67 in women [19]. Conversely, kidney stone formers also present a higher risk of developing diabetes mellitus (OR: 1.48) [20].

Experimental studies in rats have also shown a strong relationship between kidney stones, metabolic syndrome, and obesity [21]. Obesity has been shown to exacerbate oxidative stress-mediated inflammatory responses in hyperoxaluria-induced kidney stone formation. This exacerbation leads to a significant impairment of renal function and increased crystal deposition compared to rats with induced hyperoxaluria alone. The synergistic effect of obesity and hyperoxaluria results in more severe renal outcomes than hyperoxaluria by itself [22].

In our study, both a slight increase in kidney stone-related hospitalizations and an important increase in obesity coded in Spanish hospitalizations have been observed. Although these data cannot be taken as prevalence data, they reflect the trend of obesity and lithiasis prevalence in Spain since MDBS covers almost 99% of hospitalizations in our country. Between 1997 and 2021, it has been estimated that the rate of hospitalization for urinary stones was 95.6 patients per 100,000 inhabitants, varying from 76.4 from 1997 to 2001 up to 110.3 patients from 2012 to 2016. The most important data regarding the hospitality burden of kidney stones were developed in 2000 in the USA, estimating a rate of 62 cases per 100,000 inhabitants, which is slightly lower than ours for that date [23]. It is remarkable, in comparison with our data, that in the USA a negative trend in the hospitalization rate was observed, although hospital outpatients increased by 40%. It is possible that most of the procedures were being developed as an outpatient surgery without hospitalization or were accounted for differently in contrast to our country.

It is also possible to extrapolate data related to the French population in 2009 (64,710,879 inhabitants) [24] from the Raynal report, in which the hospitalization burden of lithiasis patients coded with a main diagnosis of kidney stones was estimated to be around 22.3 patients per 100,000 inhabitants, lower than ours [25].

Although it is important to compare our data to other populations, it has been shown that the impact of being overweight and obesity on stone formation is different depending on the different populations and dietary patterns. The Mediterranean diet has been shown to be associated with the same prevalence of kidney stones between normal and obese or overweight populations [26].

Our study demonstrates a strong correlation between the national hospitalization burden of kidney stones and the prevalence of obesity codes in general hospitalizations across most age groups. These findings suggest a significant relationship between the two conditions, despite the predominance of the Mediterranean diet in Spain. Notably, in populations over 70 years of age, the correlation loses statistical significance, likely due to the presence of other comorbidities associated with kidney stones in this older demographic.

To address the obesity–urolithiasis relationship in Spain, a comprehensive approach combining dietary interventions, public health campaigns, and tailored healthcare programs is recommended. Dietary measures should focus on promoting the Mediterranean diet, increasing fluid intake, and optimizing calcium consumption. Public health initiatives could include national awareness programs, school-based interventions, and media campaigns to educate the population about the link between obesity and kidney stones. This multifaceted strategy aims to reduce the prevalence of both conditions and their associated healthcare burden in Spain.

In agreement with our data, some epidemiological cross-sectional studies in Spain pointed out obesity as a risk factor for urolithiasis. The PreLiRenA study estimated the prevalence of urolithiasis at 16.4% and the incidence at 1.2% in a southern Spanish region. This study demonstrated the association of a high BMI with an OR of 1.6 [27]. Another cross-sectional study in the Spanish population, based on telephone surveys and the population aged between 40 and 69 years old, reported a prevalence of 15% and obesity/overweight augmented risk with an OR of 1.31 [7]. Another study, with a longitudinal design, and based on a single hospital database, demonstrated a significant influence of BMI on lithiasis recurrence [28]. However, no longer registers have been found using national large official databases in our country.

The data of the present study show that the obese condition multiplies by an OR of 15.6 the possibility of being hospitalized by kidney stones, in comparison to the general population. These data cannot be extrapolated to the data that estimate the risk of obesity for presenting kidney stones because the study designs are different; in our study, the data are based on hospitalization burden, and this circumstance can act as a limitation. The use of MDBS is a good method to gather information from hospitalizations in Spain since it covers practically 100% of admissions, which increases the power of the obtained results. Nevertheless, the codification in the discharge reports depends individually on the quality of this document and the information provided, and this could be a limitation. Changes in coding practices over time and evolving diagnostic criteria may influence the observed trends. Potential confounders include demographic shifts, changes in healthcare access, evolving clinical practices, and the presence of comorbidities. On the other side, the information is limited to hospitalized patients only, in contrast with the studies previously discussed in which other approaches have been used, such as telephone surveys, single-institution series, or different research networks.

Obesity and being overweight are widely known as risk factors for a number of chronic medical conditions; thus, several studies have been developed to estimate the cost or burden and the financial impact of obesity [29,30,31]. Nevertheless, as far as we know, no studies have been developed to estimate the burden of obesity code in total hospitalizations based on large national samples, and although kidney stones are widely related to obesity and being overweight, no data have been earlier reported about the impact of obesity on lithiasis disease, in terms of costs and development of the hospitalization process, as it has been reported for other diseases [32].

In our study, when comparing the length of stay between the obesity and non-obesity groups of lithiasis patients, no differences were found except for the ages between 60 and 89 years, in which the length of stay was slightly lower for the obesity group. In relation to mortality, obesity has not become a risk factor either. Non-obese lithiasis patient mortality was slightly higher than the obese group of patients, especially between the 50 and 89 age groups, when probably other comorbidities show more influence in the mortality of lithiasis patients.

Body mass index has not been related to higher mortality, although just a few studies of mortality related to kidney stone procedures have documented this feature. Obesity by itself or as part of metabolic syndrome has been shown to raise the risk of complications and other associated health conditions, which can increase the likelihood of mortality [33]. In our study, this circumstance has not been reflected, thus obese patient mortality was not influenced.

However, hospitalization costs of obese patients were significantly higher than those of non-obese patients. The obesity patient costs of hospitalization were 19.3% higher than when obesity was not coded as a comorbidity. These results are in line with those reported in other countries such as the UK, Portugal, and Brazil, in which the economic impact of the obese condition was calculated, clearly establishing that obesity represents an additional burden on healthcare systems [29,30,31]. In any of the studies reported, the specific economic burden of obesity on lithiasis patients was calculated.

Other factors such as cardiovascular disease or age have also been associated with a higher prevalence of kidney stones. Lithiasis patients develop hypertension more frequently than non-stone formers with an OR estimated between 1.24 and 1.96. Furthermore, hypertensive patients also develop a higher risk of nephrolithiasis (OR: 2.11), and hypertension has been shown as an independent factor of stone recurrence [34]. Other cardiovascular comorbidities such as myocardial infarction, coronary heart disease, and stroke are also associated with kidney stones [20,35]. In our study, the lack of a control group has not allowed us to establish the influence of hypertension or other morbidities on kidney stone incidence.

There is no updated information about Spanish prevalence of urolithiasis in the general population. A review in 2007 found an estimated incidence of 737 cases per 100,000 inhabitants a year and a prevalence of 5066 cases per 100,000 inhabitants [8]. The present project has both strengths and limitations. As mentioned previously, the use of MDBS is a good method to gather information but the codification in the discharge reports depends individually on the quality of this document and the information provided. Additionally, our study is based on the hospitalization burden; we are aware that these data cannot be directly used for general prevalence or the incidence of kidney stones or obesity. In the same way, hospital mortality rates cannot be taken as the general mortality of kidney stones or obesity since, especially in the older population, death can occur in other places rather than in hospitals (nursing homes, residences, or other institutions). The data for costs are given from 2016 to 2021 using diagnosis-related groups (DRGs), and this can also be a bias since these data have an important internal variability; nevertheless, the use of DGRs has been used widely in different scenarios to calculate hospitality costs.

## 5. Conclusions

This study reports for the first time the impact of obesity on hospitalized patients with a diagnosis of kidney stones using large data from the MBSD. The hospitalization burden of renal stones has significantly risen in the last two decades and so has the hospitalization burden of patients with obesity. A strong correlation between both conditions is shown, which suggests that obesity may be responsible for the increased prevalence of kidney stones.

Obesity significantly increases the possibility of developing kidney stones in comparison with the normal-weight population, especially in younger people, with its impact decreasing as age increases. Although obesity has no impact in terms of mortality, it represents a significant economic burden of kidney stone patients’ hospitalizations.

These data suggest that the optimal approach for the treatment of kidney stones must take into account the control of obesity and the need for establishing programs for the control of obesity especially focused on lithiasis patients.

## Figures and Tables

**Table 1:** Five-year rates of hospitalization burden of urolithiasis between 1997 and 2021 stratified by age. The data are given as a rate (95% CI) per 100,000 inhabitants.

Age	1997–2001	2002–2006	2007–2011	2012–2016	2017–2021	Overall 1997–2021	% Change	*p*
<20 years	13.3 (13–13.6)	15.5 (15.1–15.8)	16.2 (15.8–16.5)	16.9 (16.6–17.3)	8 (7.7–8.2)	14 (13.8–14.1)	−1.84	ns
20–29 years	24.6 (24–25.1)	26.1 (25.6–26.6)	28.2 (27.7–28.8)	35.3 (34.5–36)	35.8 (35–36.5)	29.4 (29.1–29.6)	2.12 (0.8–3.5)	<0.05
30–39 years	53.5 (52.7–54.3)	52.1 (51.4–52.7)	53.7 (53–54.5)	60.8 (60–61.6)	63 (62.2–64)	56.5 (56.2–56.9)	0.97 (0.06–1.9)	<0.05
40–49 years	76.1 (75–77.1)	80.9 (79.9–81.9)	85 (84.1–86)	94.4 (93.4–95.4)	95 (94–96)	87.24 (86.8–87.7)	1.2 (0.8–1.6)	<0.05
50–59 years	111.5 (110.1–112.9)	117.8 (116.4–119.1)	125.6 (124.3–127)	143.9 (142.6–145.2)	147 (145.7–148.3)	131.4 (130.8–132)	1.5 (1–2)	<0.05
60–69 years	157.1 (155.4–158.9)	171.7 (169.8–173.5)	178.8 (177–180.6)	199.7 (197.9–201.5)	195.2 (193.5–196.9)	181.9 (181.1–182.7)	1.2 (0.5–1.8)	<0.05
70–79 years	214.3 (211.2–216.7)	253.1 (250.7–255.5)	258.5 (256.1–260.9)	272.3 (269.8–274.8)	231.5 (229.4–233.7)	246.5 (245.5–247.6)	0.5 (−0.8–1.7)	ns
80–89 years	238.8 (234.8–242.9)	318.3 (314.1–322.5)	348.3 (344.4–352.3)	359.2 (355.6–362.9)	257.1 (254.1–260.2)	307.9 (306.3–309.6)	0.5 (−1.7–2.8)	ns
>89 years	231.1 (220.3–242.2)	315.1 (304–326.5)	342 (331.7–352.5)	351.6 (342.9–360.6)	246.3 (239.9–252.8)	297.2 (293.1–301.2)	0.48 (−1.8–2.8)	ns
Total	76.4 (76–76.8)	87.8 (87.4–88.2)	95.2 (94.8–95.6)	110.3 (109.9–110.8)	105.3 (104.9–105.7)	95.6 (95.4–95.8)	1.8 (0.2–3.3)	<0.05

**Table 2:** Five-year rates of hospitalization burden of obesity between 1997 and 2021 stratified by age. The data are given as a rate (95% CI) per 100,000 inhabitants.

Age	1997–2001	2002–2006	2007–2011	2012–2016	2017–2021	Overall 1997–2021	% Change	*p*
<20 years	3.8 (3.6–4)	4.6 (4.4–4.8)	9.7 (9.4–10)	17.9 (17.5–18.3)	28 (27.6–28.5)	12.9 (12.8–13.1)	11.3 (8.5–14.1)	<0.01
20–29 years	7.7 (7.5–8)	12.3 (11.9–12.7)	32.8 (32.1–33.3)	78.8 (77.7–79.9)	128.2 (126.8–129.6)	45.9 (45.5–46.2)	16.1 (11.4–20.9)	<0.001
30–39 years	21 (20.5–21.5)	34.1 (33.5–34.7)	71.5 (70.7–72.3)	143.8 (142.5–145)	240.9 (239.2–242.6)	100.3 (99.8–100.8)	13.5 (11.7–15.2)	<0.001
40–49 years	55.5 (55–56.4)	88.2 (87.1–89.2)	150.4 (149.1–151.7)	227.9 (226.4–229.4)	300.8 (299–302.5)	176.2 (175.5–176.8)	9 (6.2–12)	<0.001
50–59 years	123.8 (122.3–125.3)	215 (213.2–216.9)	340.4 (338.3–342.6)	471.4 (469–473.8)	583.7 (581.2–586.3)	373.8 (372.7–374.8)	8.1 (5.8–10.4)	<0.01
60–69 years	222.6 (220.5–224.7)	407.7 (404.9–410.5)	655.26 (651.8–658.7)	905.6 (901.7–909.4)	1064.7 (1060.8–1068.7)	681.8 (680.3–683.4)	8.2 (5.6–10.8)	<0.01
70–79 years	273.49 (270.8–276.2)	602.7 (598.9–606.4)	1084.7 (1079.8–1089.6)	1522.4 (1516.5–1528.3)	1695.7 (1689.8–1701.6)	1070.5 (1068.3–1072.7)	9.6 (5.4–13.8)	=0.01
80–89 years	208.4 (204.7–212.2)	538 (532.5–543.5)	1162.1 (1154.9–1169.3)	1853.8 (1845.5–1862.2)	2223.5 (2214.7–2232.4)	1374.2 (1370.7–1377.7)	12.7 (6.8–18.7)	<0.01
>89 years	128.2 (120.2–136.6)	335.5 (324–347.2)	751.4 (736.1–767)	1287.8 (1270.9–1304.8)	1708.5 (1691.6–1725.5)	1075.5 (1067.8–1083.3)	13.9 (8.6–19.4)	<0.01
Total	74.5 (74.1–74.9)	147.8 (147.3–148.3)	271.58 (270.8–272.1)	430.2 (429.3–431)	557.9 (557–558.9)	305.2 (304.9–305.5)	10.7 (7.3–14.2)	<0.01

**Table 3:** Hospitalization rates (rate per 100,000 inhabitants) of lithiasis diagnoses related to general population and obesity hospitalizations in Spain by age (1997–2021). Data are given as rate (95% CI) per 100,000 inhabitants.

Age	Lithiasis in General Population	Lithiasis in Obesity Population	Odds Ratio	*p*
<20 years	12.3 (12.2–12.5)	569.2 (488–660.1)	46.4 (39.9–53.8)	*p* < 0.0001
20–29 years	25.9 (25.7–26.2)	691.6 (631.2–756.2)	26.9 (24.5–29.4)	*p* < 0.0001
30–39 years	51.4 (51.1–51.7)	1126.9 (1078.4–1177)	22.2 (21.2–23.2)	*p* < 0.0001
40–49 years	80.3 (79.9–80.7)	1712 (1665.7–1759.1)	21.7 (21.1–22.3)	*p* < 0.0001
50–59 years	120.8 (120.2–121.3)	1730.5 (1695.2–1766.3)	14.6 (14.3–14.9)	*p* < 0.0001
60–69 years	163.8 (163.1–164.5)	1523.4 (1496–1551.2)	9.42 (9.3–9.6)	*p* < 0.0001
70–79 years	225.8 (224.8–226.7)	1213.6 (1191.1–1236.3)	5.4 (5.3–5.5)	*p* < 0.0001
80–89 years	286.6 (285–288.1)	975.1 (950–1000.7)	3.4 (3.3–3.5)	*p* < 0.0001
>89 years	282.9 (279.1–286.8)	639.9 (583.1–698.6)	2.3 (2.1–2.5)	*p* < 0.0001
Total	86.4 (86.2–86.6)	1330.5 (1318.8–1342.7)	15.6 (15.4–15.7)	*p* < 0.0001

**Table 4:** Impact of obesity on mortality (%), length of stay (days), and costs (EUR) in Spain (1997–2021). Data of costs are only given from 2016 to 2021. ns = non significant.

Age	Length of Stay $\mathrm{Length}\;\mathrm{of}\;\mathrm{Stay}\;\bar{x}$ (95% CI) Days			Mortality % (95% CI)			Cost $\mathrm{Length}\;\mathrm{of}\;\mathrm{Stay}\;\bar{x}$ (95% CI) EUR		
	Normal Weight	Obesity	*p*	Normal Weight	Obesity	*p*	Normal Weight	Obesity	*p*
<20 years	7 (6.9–7.1)	7.3 (5.5–9.1)	ns	0.3 (0.3–0.4)	0.6 (0.01–3.2)	ns	3379 (3267–3490)	3540 (3117–3964)	ns
20–29 years	5.2 (5.1–5.3)	5 (4.4–5.5)	ns	0.2 (0.1–0.2)	0.6 (0.1–0.2)	ns	3085 (3040–3129)	3638 (3387–3890)	*p* < 0.0001
30–39 years	5.24 (5.1–5.4)	5.7 (5.3–6)	ns	0.4 (0.3–0.4)	0.3 (0.1–0.6)	ns	3128 (3098–3157)	3963 (3760–4166)	*p* < 0.0001
40–49 years	5.6 (5.4–5.8)	5.7 (5.5–5.9)	ns	0.7 (0.6–0.7)	0.8 (0.6–1.1)	ns	3383 (3357–3408)	4344 (4176–4511)	*p* < 0.0001
50–59 years	6.2 (6.1–6.2)	6.4 (6.2–6.6)	0.01	1.3 (1.2–1.4)	0.9 (0.8–1.2)	*p* = 0.005	3831 (3800–3862)	4721 (4601–4841)	*p* < 0.0001
60–69 years	7.7 (7.7–7.8)	7.3 (7.1–7.5)	<0.001	2.4 (2.4–2.5)	1.7 (1.5–2)	*p* < 0.0001	4318 (4282–4354)	5070 (4950–5190)	*p* < 0.0001
70–79 years	9.5 (9.4–9.5)	8.7 (8.5–8.9)	<0.001	4.6 (4.5–4.7)	3.8 (3.4–4.1)	*p* < 0.0001	4760 (4719–4801)	5100 (4971–5228)	*p* < 0.0001
80–89 years	10 (10–10.1)	9.3 (9.1–9.5)	<0.001	9 (8.8–9.1)	6.7 (6–7.3)	*p* < 0.0001	4763 (4717–4807)	4860 (4709–5010)	ns
>89 years	9.6 (9.4–9.7)	9.2 (8.5–9.9)	ns	14.9 (14.4–15.3)	13.4 (10.5–16.8)	ns	4628 (4544–4712)	4856 (4376–5337)	ns
Total	7.4 (7.4–7.5)	7.5 (7.4–7.6)	ns	3 (3–3.1)	2.6 (2.4–2.7)	*p* < 0.001	4015 (4002–4030)	4789 (4732–4846)	*p* < 0.0001

## Data Availability

The datasets generated during and/or analyzed during the current study are available from the corresponding author upon reasonable request.

## References

[1] Ramello A., Vitale C., Marangella M. (2000). Epidemiology of nephrolithiasis. J. Nephrol..

[2] Trinchieri A. (2008). Epidemiology of urolithiasis: An update. Clin. Cases Miner. Bone Metab..

[3] Romero V., Akpinar H., Assimos D.G. (2010). Kidney stones: A global picture of prevalence, incidence, and associated risk factors. Rev. Urol..

[4] Di Cesare M., NCD Risk Factor Collaboration (2024). Worldwide trends in underweight and obesity from 1990 to 2022: A pooled analysis of 3663 population-representative studies with 222 million children, adolescents, and adults. Lancet.

[5] Taylor E.N., Fung T.T., Curhan G.C. (2009). DASH-style diet associates with reduced risk for kidney stones. J. Am. Soc. Nephrol..

[6] Saenz-Medina J., Muñoz M., Rodriguez C., Contreras C., Sánchez A., Coronado M.J., Ramil E., Santos M., Carballido J., Prieto D. (2022). Hyperoxaluria Induces Endothelial Dysfunction in Preglomerular Arteries: Involvement of Oxidative Stress. Cells.

[7] Saenz-Medina J., Muñoz M., Rodriguez C., Contreras C., Sánchez A., Coronado M.J., Ramil E., Santos M., Carballido J., Prieto D. (2017). Comorbidity and sociodemographic factors associated with renal lithiasis in persons aged 40 to 65: A cross-sectional study. Med. Clin..

[8] Sánchez-Martín F.M., Martínez-Rodríguez R. (2007). Incidence and prevalence of published studies about urolithiasis in Spain. A review. Actas Urol. Esp..

[9] De Lorenzo A., Gratteri S., Gualtieri P., Cammarano A., Bertucci P., Di Renzo L. (2019). Why primary obesity is a disease?. J. Transl. Med..

[10] Grases F. (2017). Epidemiology of renal lithiasis and associated factors. Med. Clin..

[11] Sorokin I., Mamoulakis C., Miyazawa K., Rodgers A., Talati J., Lotan Y. (2017). Epidemiology of stone disease across the world. World J. Urol..

[12] Levy D.T., Mabry P.L., Wang Y.C., Gortmaker S., Huang T.K., Marsh T., Moodie M., Swinburn B. (2011). Simulation models of obesity: A review of the literature and implications for research and policy. Obes. Rev..

[13] Manzoor M.A.P., Agrawal A.K., Singh B., Mujeeburahiman M., Rekha P.-D. (2019). Morphological characteristics and microstructure of kidney stones using synchrotron radiation μCT reveal the mechanism of crystal growth and aggregation in mixed stones. PLoS ONE.

[14] Grases F., Costa-Bauzá A., Prieto R.M. (2017). May renal lithiasis be really prevented? New trends and therapeutic tools. Arch. Esp. Urol..

[15] Khan S.R., Pearle M.S., Robertson W.G., Gambaro G., Canales B.K., Doizi S., Traxer O., Tiselius H.G. (2016). Kidney stones. Nat. Rev. Dis. Primers.

[16] Arbelaez M.C.S., Nackeeran S., Shah K., Blachman-Braun R., Bronson I., Towe M., Bhat A., Marcovich R., Ramasamy R., Shah H.N. (2023). Association between body mass index, metabolic syndrome and common urologic conditions: A cross-sectional study using a large multi-institutional database from the United States. Ann. Med..

[17] West B., Luke A., Durazo-Arvizu R.A., Cao G., Shoham D., Kramer H. (2008). Metabolic syndrome and self-reported history of kidney stones: The National Health and Nutrition Examination Survey (NHANES III) 1988-1994. Am. J. Kidney Dis..

[18] Carbone A., Al Salhi Y., Tasca A., Palleschi G., Fuschi A., De Nunzio C., Bozzini G., Mazzaferro S., Pastore A.L. (2018). Obesity and kidney stone disease: A systematic review. Minerva Urol. Nefrol..

[19] Taylor E.N., Stampfer M.J., Curhan G.C. (2005). Diabetes mellitus and the risk of nephrolithiasis. Kidney Int..

[20] Domingos F., Serra A. (2011). Nephrolithiasis is associated with an increased prevalence of cardiovascular disease. Nephrol. Dial. Transplant..

[21] Saenz J. (2016). Murine model for the evaluation of hyperoxaluria on metabolic syndrome patients. Eur. Urol. Suppl..

[22] Sáenz-Medina J., Muñoz M., Sanchez A., Rodriguez C., Jorge E., Corbacho C., Izquierdo D., Santos M., Donoso E., Virumbrales E. (2019). Nox1-derived oxidative stress as a common pathogenic link between obesity and hyperoxaluria-related kidney injury. Urolithiasis.

[23] Pearle M.S., Calhoun E.A., Curhan G.C., Urologic Diseases of America Project (2005). Urologic diseases in America project: Urolithiasis. J. Urol..

[24] World Bank Open Data. https://data.worldbank.org.

[25] Raynal G., Merlet B., Traxer O. (2011). In-hospital stays for urolithiasis: Analysis of French national data. Prog. Urol..

[26] Trinchieri A., Croppi E., Montanari E. (2017). Obesity and urolithiasis: Evidence of regional influences. Urolithiasis.

[27] Cano-Castiñeira R., Carrasco-Valiente J., Pérula-de-Torres L.A., Jiménez-García C., Olaya-Caro I., Criado-Larumbe M., Requena-Tapia M.J. (2015). Prevalence of renal stones in Andalusian population: Results of PreLiRenA study. Actas Urol. Esp..

[28] Sáenz J., Páez A., Alarcón R.O., Casas J.M., Sánchez A., Pereira E., Cáncer E., Alvarez M., Rendón D., Durán M. (2012). Obesity as risk factor for lithiasic recurrence. Actas Urol. Esp..

[29] Bahia L., Coutinho E.S.F., Barufaldi L.A., de Azevedo Abreu G., Malhão T.A., Ribeiro de Souza C.P., Araujo D.V. (2012). The costs of overweight and obesity-related diseases in the Brazilian public health system: Cross-sectional study. BMC Public Health.

[30] Allender S., Rayner M. (2007). The burden of overweight and obesity-related ill health in the UK. Obes. Rev..

[31] Destri K., Henriques A.R., Mendonça N., Alves J., Barcelos A., Dias S.S., Gregório M.J., Canhão H., Rodrigues A.M. (2024). Hospitalization costs in Portugal among people with obesity: Results from a nationwide population-based cohort 2011 to 2021. Front. Public Health.

[32] Javaid S., Frasier K., Chaudhary A.J. (2024). Impact of obesity on in-hospital mortality and morbidity among patients admitted for antineoplastic chemotherapy: A nationwide analysis. Clin. Transl. Oncol..

[33] Bhanot R., Pietropaolo A., Tokas T., Kallidonis P., Skolarikos A., Keller E.X., De Coninck V., Traxer O., Gozen A., Sarica K. (2022). Predictors and Strategies to Avoid Mortality Following Ureteroscopy for Stone Disease: A Systematic Review from European Association of Urologists Sections of Urolithiasis (EULIS) and Uro-technology (ESUT). Eur. Urol. Focus..

[34] Kim F., Pham M., Maloney E., Rizzo N.O., Morton G.J., Wisse B.E., Kirk E.A., Chait A., Schwartz M.W. (2008). Vascular inflammation, insulin resistance, and reduced nitric oxide production precede the onset of peripheral insulin resistance. Arterioscler. Thromb. Vasc. Biol..

[35] Saenz-Medina J., Muñoz M., Rodriguez C., Sanchez A., Contreras C., Carballido-Rodríguez J., Prieto D. (2022). Endothelial Dysfunction: An Intermediate Clinical Feature between Urolithiasis and Cardiovascular Diseases. Int. J. Mol. Sci..

